# Toward the Definition of a Soundscape Ranking Index (SRI) in an Urban Park Using Machine Learning Techniques

**DOI:** 10.3390/s23104797

**Published:** 2023-05-16

**Authors:** Roberto Benocci, Andrea Afify, Andrea Potenza, H. Eduardo Roman, Giovanni Zambon

**Affiliations:** 1 Department of Earth and Environmental Sciences (DISAT), University of Milano-Bicocca, Piazza della Scienza 1, 20126 Milano, Italy; a.potenza@campus.unimib.it (A.P.); giovanni.zambon@unimib.it (G.Z.); 2 Department of Physics, University of Milano-Bicocca, Piazza della Scienza 3, 20126 Milano, Italy; a.afify@campus.unimib.it; 3 NEXiD Edge, NEXiD, Via Fabio Filzi 27, 20124 Milano, Italy

**Keywords:** soundscape, ecoacoustic indices, soundscape ranking index (SRI), urban parks, machine learning

## Abstract

The goal of estimating a soundscape index, aimed at evaluating the contribution of the environmental sound components, is to provide an accurate “acoustic quality” assessment of a complex habitat. Such an index can prove to be a powerful ecological tool associated with both rapid on-site and remote surveys. The soundscape ranking index (SRI), introduced by us recently, can empirically account for the contribution of different sound sources by assigning a positive weight to natural sounds (biophony) and a negative weight to anthropogenic ones. The optimization of such weights was performed by training four machine learning algorithms (decision tree, DT; random forest, RF; adaptive boosting, AdaBoost; support vector machine, SVM) over a relatively small fraction of a labeled sound recording dataset. The sound recordings were taken at 16 sites distributed over an area of approximately 22 hectares at Parco Nord (Northern Park) of the city Milan (Italy). From the audio recordings, we extracted four different spectral features: two based on ecoacoustic indices and the other two based on mel-frequency cepstral coefficients (MFCCs). The labeling was focused on the identification of sounds belonging to biophonies and anthropophonies. This preliminary approach revealed that two classification models, DT and AdaBoost, trained by using 84 extracted features from each recording, are able to provide a set of weights characterized by a rather good classification performance (F1-score = 0.70, 0.71). The present results are in quantitative agreement with a self-consistent estimation of the mean SRI values at each site that was recently obtained by us using a different statistical approach.

## 1. Introduction

Among the elements used to evaluate the environmental status as a whole, one is strictly connected to the acoustic quality of a habitat, recognized as a vital dimension of wildlife conservation [1,2]. The induced modifications prompted by the encroaching urbanization with increasingly excessive human noise and a lack of gradients between natural and built environments can lead to direct deleterious effects on biodiversity as documented in recent works [3,4,5,6].

The diffusion of passive acoustic monitoring with a large memory capability, and the possibility of analyzing acoustic recordings by extracting specific spectral and level characteristics through ecoacoustic indices (see [7] for a review), allow us to retrieve important information about the unique assemblage of sounds across space and time. Such habitat characteristics are collectively referred to as a soundscape [8,9], the latter recognized as a distinct feature or ecological “signature” of a landscape [10,11].

Such characteristics can indeed be reflected in ecoacoustics indices calculated over predefined time intervals. Thus, they integrate the acoustic dynamics of an ecosystem, consisting of vocalizing species, anthropogenic noise, and natural phenomena [12], into a set of time series that can be proved to explain observed changes in habitat status [13], providing insights on species diversity and human impacts across a wide range of terrestrial [14,15,16] and aquatic environments [17,18]. The validation of ecoacoustic indices calculation is usually sound-truthed by specialized operators that classify hours of recordings according to predefined sound categories.

This identification procedure of sound sources is highly time consuming and requires specific knowledge of animal vocalizations. This necessarily limits its applicability to small datasets [19,20]. A cumulative approach that provides a qualitative description of the recorded sound (e.g., many/few vocalizing birds, many/few birds species, high/low traffic noise, etc.) partially improved the validation process, showing good matching between the “manual” identification of acoustic categories and ecoacoustic indices [21]. Thus, the need for employing unsupervised methods to process large amounts of information, independently of human intervention, is evident. Having access to such techniques can allow us to study huge datasets on very different time and spatial scales, prompting the disentangling of information hidden within the complex network of interest. To this end, we resort to machine learning (ML) techniques. The latter are currently widely used to train models using empirical data for a plethora of applications, such as translation, text classification, web searching, image recognition, and speech recognition. For instance, some of the first relevant applications were developed for the classification of handwritten digits [22] and the automatic composition of music [23,24].

It is widely recognized that ML techniques have the ability to learn generic spatial features [25,26], suitable in particular for image-related tasks. Recent applications of deep learning (DL) and ML computations for studying soundscape dynamics show promising results in terms of species identification [27,28], the separation of audio sources by using a set of techniques aimed at recovering individual sound sources when only mixtures are accessible [29], and also unsupervised classifications by means of convolutional neural networks (CNNs) [11]. In urban areas, ML models have been applied for predicting long-term acoustic patterns from short-term sound pressure level measurements [30] and for detecting anomalous noise sources prior to computing traffic noise maps [31]. CNNs have been applied to soundscape classification [32,33], species-specific recognition [34,35,36], and the identification of multiple and simultaneous acoustic sources using a two-stage classifier able to determine, in real time, simultaneous urban acoustic events taking advantage of physical redundancy from a wireless acoustic sensors network (WASN) in the city of Barcelona [37].

Several soundscape sources have been classified using two CNN classifiers to distinguish between biophony and anthropophony in the city of London by training CNN models on a limited quantity of labeled sound samples [28]. Their results exceed the analysis performed by multiple acoustic indices. Other attempts successfully provided sound sources identification at the price of a huge “manual” procedure in approximately 60,000 sound recordings [38]. The prediction of soundscape components, including quiet periods and microphone interference, was also performed by training a CNN with a huge dataset collected over four years across Sonoma County (California) by citizen scientists with high precision [39].

Other examples of ML applications to soundscape prediction can be found in [40,41,42,43,44]. In [40], the authors present a method for the automatic recognition of the soundscape quality of urban recordings by applying four different support vector machine (SVM) regressors to a combination of spectral features. In [41], a mixture of features (temporal, spectral, and perceptual) was used to classify urban sound events belonging to nine different categories. In [42], the detection and classification of acoustic events were obtained by using a modified Viterbi decoding process in combination with weighted finite-state transducers (WFSTs). In [43], acoustic indicators collected from the city of Barcelona were used to train several clustering algorithms, demonstrating the possibility of parceling the city based on the noise levels in the area. In [44], an unsupervised learning technique was applied to group the nodes of a WASN in clusters with the same behavior, thus recognizing complex patterns on this basis. Other studies make use of ML techniques together with signal processing to classify acoustic events at subsequent stages (layers) [45].

In [46], two types of supervised classifiers, namely artificial neural networks (ANNs) and a Gaussian mixture model (GMM), were compared to determine the primary noise source in the acoustic environment. In [47], the authors combined local features and short-term sound recording features with long-term descriptive statistics to create a deep convolutional neural network for classifying urban sound events. In [48], four well-known deep neural networks were fine-tuned for bird species classification.

As can be appreciated, the use of ML techniques have mostly found applications in soundscape studies by correlating different noise events to the perception of the population, with the aim of automatically detecting potentially disturbing noise events (see also [49]). When using traditional ML algorithms, the choice of appropriate features of the audio file, either in the form of frequency content, dynamic information, or both, always represents the first step in the analysis. In this regard, the most widely used feature is represented by the mel-frequency cepstral coefficient (MFCC).

Urban parks represent a unique area of study as retrieving source-specific information from geophony, biophony, and anthropophony remains a challenging task due to interference and confounding factors arising from the simultaneous presence of different sound sources. It should be emphasized that, in the existing literature, the question of defining an indicator that ‘summarizes’ the information about the acoustic environment unbound from human perceptual nature has not generally been considered. In order to fill this gap, we devised an index enabling us to quantify the quality of the local environment sound in a simple fashion. The index is referred to as the soundscape ranking index (SRI), and is assembled by weighting the different soundscape components (geophony, biophony, and anthropophony) present in a habitat/environment. The identification of the different soundscape components requires a time-intensive “manual” labeling or sound truthing of the recorded audio files, and is usually performed by a single expert.

In this work, we studied the possibility of predicting the SRI at an urban park in the city of Milan (Italy) from the extracted spectral features of audio recordings. This task was pursued by applying a set of ML classification models to our dataset collected over an extended area, where the resulting indexes were grouped, for simplicity and in accord with our previous works, into three main categories denoted as “poor”, “intermediate”, and “good” environment sound qualities. This classification was obtained according to the different contributions of the environment sound sources. These groups are influenced by the choice of the set of weights attributed to each soundscape component (typically the weight is given a positive value in the case of the presence of biophonies and a negative value in the presence of anthropophonies). Here, we used the classification capabilities of the chosen ML algorithms to fine-tune the soundscape weights, thus obtaining the optimal separation of the area of study in terms of local environmental sound qualities. The use of ML techniques to study the SRI will allow us to consider much larger datasets than those studied by means of supervised methods requiring human intervention. Work along this line is in progress.

The paper is organized as follows. Section 2 describes the area of study and the instrumentation used. The formulation of SRI in terms of weighting the different soundscape components is discussed together with the ML optimization procedure used. The classification models and spectral features representing the dataset are described in detail. The results are presented in Section 3, where the different models are also discussed on the basis of the validation procedures. In Section 4, we summarize the main achievements of the present work and outline possible future developments along the present lines.

## 2. Materials and Methods

In this section, we briefly describe the area of study, instrumentation, and recording pattern. We include the description of the SRI index, the scheme used for its prediction and optimization based on the features extracted from the spectral analysis of audio recordings in the form of ecoacoustic indices and mel-frequency cepstral coefficients (MFCCs), and the manual labeling. We also illustrate the classification models used to predict the manual labeling from the extracted features of the audio recordings.

### 2.1. Area of the Study

**Figure 1:**
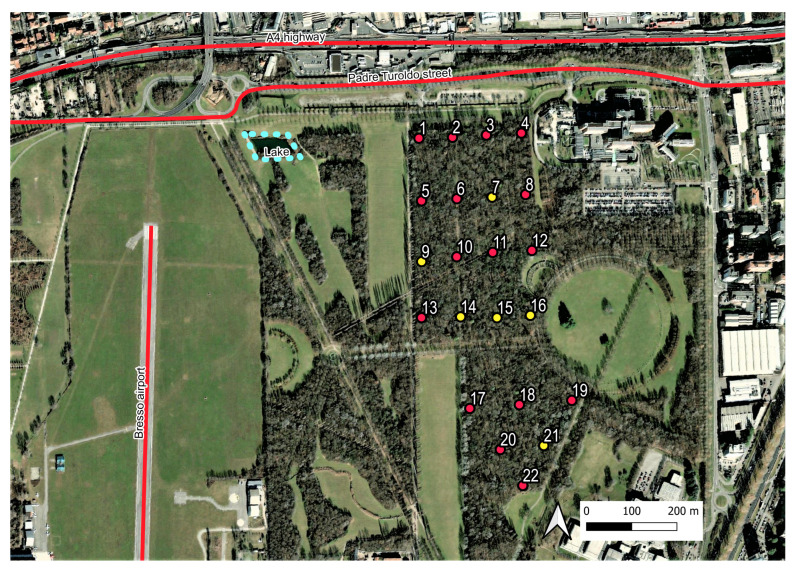
Area surrounding the grid of sensors indicated as numbered spots. Red spots indicate the active recording sites, and yellow spots indicate sites with recording disruption. In the figure, the A4 highway, Padre Turoldo Street, and Bresso airport runaway are indicated.

The Parco Nord (Northern Park) in the city of Milan extends over an area of approximately 790 hectares and is located within a highly urbanized area. Approximately 45% of its surface is dedicated to natural green spaces and vegetation, whereas the remaining surface is devoted to agricultural activities and infrastructures. The area of study is a tree-covered parcel of approximately 22 hectares encircled by agricultural fields, lawns, paths, and roads (see Figure 1). It has a semi-natural structure that is characterized by herbaceous layers of nemoral flora, shrub and arboreal layers, and dead wood. The area is crossed by numerous paths and is mainly used for recreational activities. It contains an artificial lake of approximately 300 m${}^{2}$ located approximately 250 m from the edge of the bush. The main traffic noise sources are the A4 highway and the Padre Turoldo street, located to the north at around 100 m from the wooded parcel. There is also the presence of a small airport (Bresso airport) on the west side at around 500 m from the tree-line edge.

### 2.2. Audio Recorders

**Figure 2:**
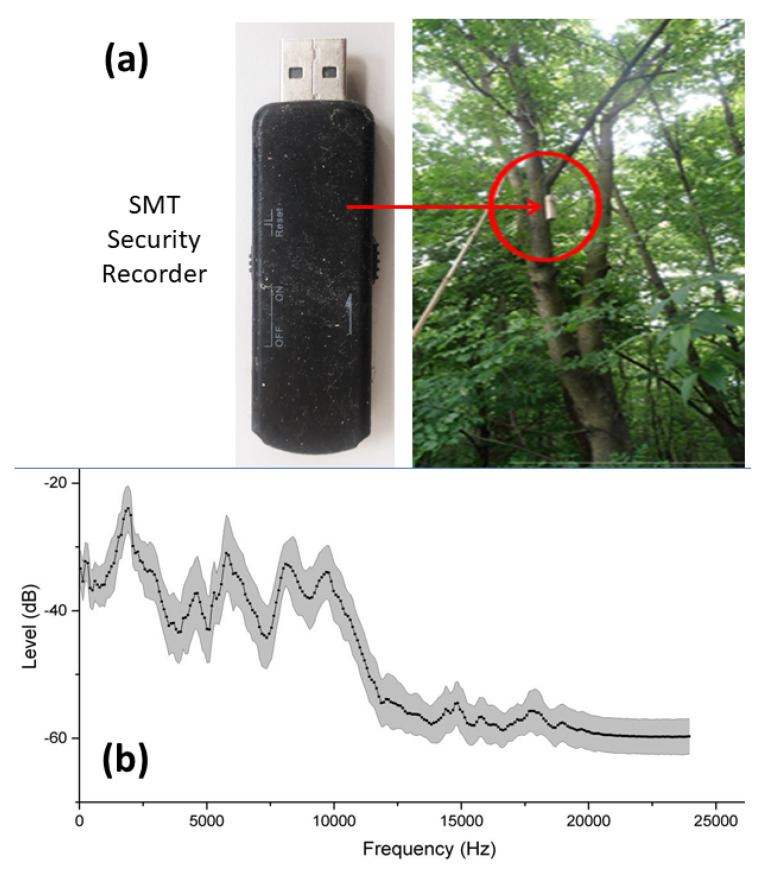
(**a**) SMT Security recorder and its practical location (red circle) on a tree. (**b**) Average spectrum (computed using a 512-point FFT) of sound level response of the recorders [dB] vs. frequency in the range (0–24) kHz. The gray band around the curve corresponds to one standard deviation of the response calculated over several SMT Security recorders. Sensitivity decreases for frequencies higher than 10 kHz.

We used low-cost digital audio recorders produced by the SMT Security (Figure 2a). They were set to measure continuously with a sampling rate of 48 kHz and were equipped with a two-week lifetime powerbank. Before using low-cost devices, it was necessary to verify their possible different frequency responses in the frequency range of interest. Thus, initial tests were devoted to selecting those recorders with a frequency response within 5% of the average spectrum calculated over all recorders. The average spectrum was computed using a 512-point FFT analysis by applying a white noise as a sound source. Full details of the the frequency characterization of the recorders can be found in [50]. The results are reported in Figure 2b, where a reduction in sensitivity for frequencies higher than 10 kHz is observed.

### 2.3. Measurement Scheme

The 22 recorders were initially positioned on a regular grid, as shown in Figure 1, covering an area of approximately 270 × 500 m${}^{2}$, plus another grid with an area of 300 × 250 m${}^{2}$ for the southern part of the parcel. The recordings were scheduled for the period of greatest singing activity of the avifauna and repeated over four days, namely on 25–28 May 2015, from 06:30 a.m. to 10:00 a.m. (CET), corresponding to 3.5 h for each site and for each recording session. Unfortunately, six recorders did not work properly (see yellow spots in Figure 1) and thus the audio files analyzed in this study involved only 16 sites.

### 2.4. Aural Survey

**Figure 3:**
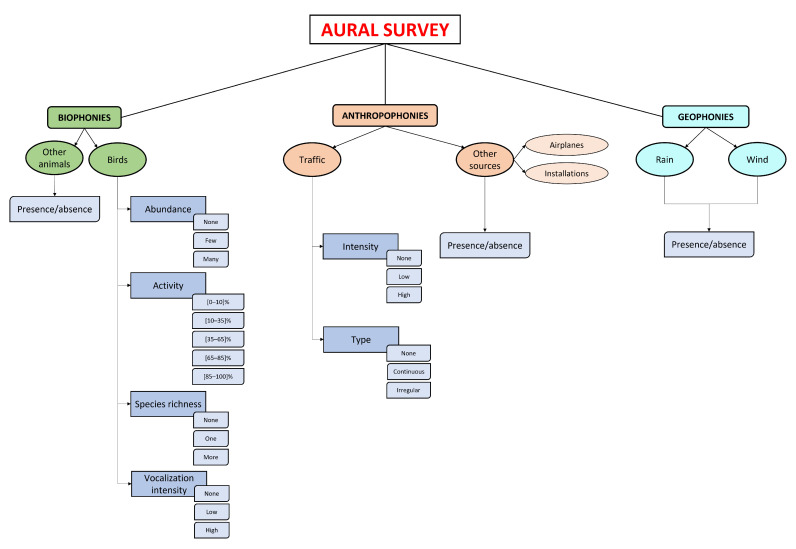
Classification of sound sources considered for the aural survey corresponding to six categories: birds, other animals, road traffic noise, other noise sources (airplanes, trains), rain, and wind. For each category, the following attributes were considered. Birds: (1) individual abundance, (2) perceived singing activity (%), (3) species richness, (4) vocalization intensity. Other biological sound sources: presence–absence. Anthropogenic noise: (1) noise intensity, (2) typology of traffic. Other anthropogenic sources: presence–absence. Geophonies were absent in the considered recordings.

In this section, we describe the scheme adopted for the aural analysis of audio files in order to quantify distinctive sound features. An aural survey was carried out to quantify the biophonies, anthropophonies, and geophonies. In particular, a single expert listened carefully to the recordings according to the following scheme: one minute listened to for every three minutes of continuous recording, for a total of seventy minutes of listening per site. The expert focused on quantifying the biophonic activity (mainly avian vocalizations) and the tecnophonic sources, evaluating the parameters reported in the scheme of Figure 3.

Each soundscape component was analyzed to extract information about the sound source and its occurrence and intensity (see Figure 3). Following this criterion, the avian vocalizations were the most studied. For each minute listened, four parameters were evaluated: (1) individual abundance (no–few–many subjects), (2) perceived singing activity expressed as the percentage of time occupied by avian vocalizations (0–100%), (3) species richness (none–one–more than one species), and (4) vocalization intensity (no–low–high intensity). For other biological sound sources, such as other animals and people, just the presence–absence indicator was used.

The anthropogenic noise is mainly attributable to road traffic. For this source, two parameters were evaluated (see Figure 3): (1) noise intensity (no–low–high intensity) and (2) the typology of traffic (no–continuous–intermittent traffic). Other sources, such as installations and airplanes, were also studied using the presence–absence indicator. Finally, given their poor contribution to the soundscape of the area during the measurement campaign, geophonies such as rain and wind were not considered.

### 2.5. The Soundscape Ranking Index, SRI

We wish to quantify the quality of the local environment sound by means of a single index, SRI, as proposed recently [51]. In the following, we briefly recall the definition of the SRI, introduced to describe, on average, the local environment sound,

(1) $${\mathrm{SRI}}_{N_{T}}=\frac{1}{N_{T}}\sum_{r=1}^{N_{T}}\sum_{i=0}^{n_{c}}c_{N_{i}}\;N_{i,r},$$

where $N_{T}$ refers to the total number of recordings, $n_{c}+1$ is the total number of identified categories (birds, other animals, road traffic noise, other noise sources, and rain and wind)—here, $n_{c}=4$ and $N_{i,r}=1$ if the *i*th sound category is present at the recording *r*; otherwise, $N_{i,r}=0$—and $c_{N_{i}}$s are coefficients chosen within the ranges displayed in Table 1 [51].

In the present study, a single audio recording, i.e., $N_{T}=1$, was considered. The reason for this choice relies on the need to compare the present new results with those discussed by us in a previous work [52]. It can also be seen as providing a “snapshot” of the local soundscape. It should be emphasized that the present work is a first attempt to estimate the SRI for the Northern Park in Milan using ML techniques. Extensions of this analysis to more audio recordings is under consideration and the results will be considered elsewhere. Each set of calculated spectral features is expected to be correlated to a series of manually recognized sound categories within the single audio recording (we can provide the audio recording data upon request). In this specific case, Equation (1) becomes (see also [52])

(2) $${\mathrm{SRI}}_{\ell }=\sum_{i=0}^{n_{c}}c_{N_{i}}\;N_{i,\ell },$$

where the subindex *ℓ* refers to the *ℓ*th recording and the coefficients take on the following values: $c_{N_{i}}>0$ ($c_{N_{i}}\to$$c_{+}$, $c_{++}$) when a sound category is associated with a natural sound, where we have split the values into two subranges (+, ++), and $c_{N_{i}}<0$ ($c_{N_{i}}\to$$c_{-}$, $c_{--}$) for a potential disturbing event, also split into two subranges. The absence of bird vocalization is regarded as neutral, $c_{N_{0}}=0$. In Table 1, we report the assumed ranges of variability for $c_{N_{i}}$. Note that in [52] we used the notation $P\left(1\right)=c_{++}$, $P\left(2\right)=c_{+}$, $P\left(3\right)=c_{N_{0}}$, $P\left(4\right)=c_{-}$, and $P\left(5\right)=c_{--}$.

Thus, Equation (2) provides a number that is expected to be representative of the environmental sound quality. Following our previous works [51,52], we chose three intervals of the SRI to define the environmental sound quality, for a single recording denoted simply as *ℓ*, given by

(3) $$\begin{array}{ccc} {\mathrm{SRI}}_{\ell }\; & & <0\;\left[\mathrm{poor}\;\;\mathrm{quality}\right],\\ 0\;\;\leq \;\;{\mathrm{SRI}}_{\ell }\; & & \leq 2\;\left[\mathrm{medium}\;\;\mathrm{quality}\right],\\ {\mathrm{SRI}}_{\ell }\; & & >2\;\left[\mathrm{good}\;\;\mathrm{quality}\right]. \end{array}$$

It should be emphasized that the choice of these intervals for classifying the SRI is rather arbitrary. Nonetheless, they are based on rather generic features attributed to human perception, in the sense that the poor (good) quality interval reflects a prevalence of anthropogenic (biophonic) sound sources, whereas the intermediate quality interval reflects a balance/co-existence of both types of sound sources.

### 2.6.  SRI Optimization Procedure

We first searched for the sound categories, reported in Table 2, by manually labeling the audio recording (see [52] for additional details). Each category *i* was assigned a weight, $c_{N_{i}}$, according to the attributes present in the audio recording (see column ‘Attribute’ in Table 2). The singing activity was assigned a weight that depends on the percentage of birds singing in each recording, e.g., for a singing activity in the interval (0, 25]%, we assigned a weight of $0.25\times c_{++}$, whereas, for (25, 50]%, we assigned a weight of $0.50\times c_{++}$, etc.

**Figure 4:**
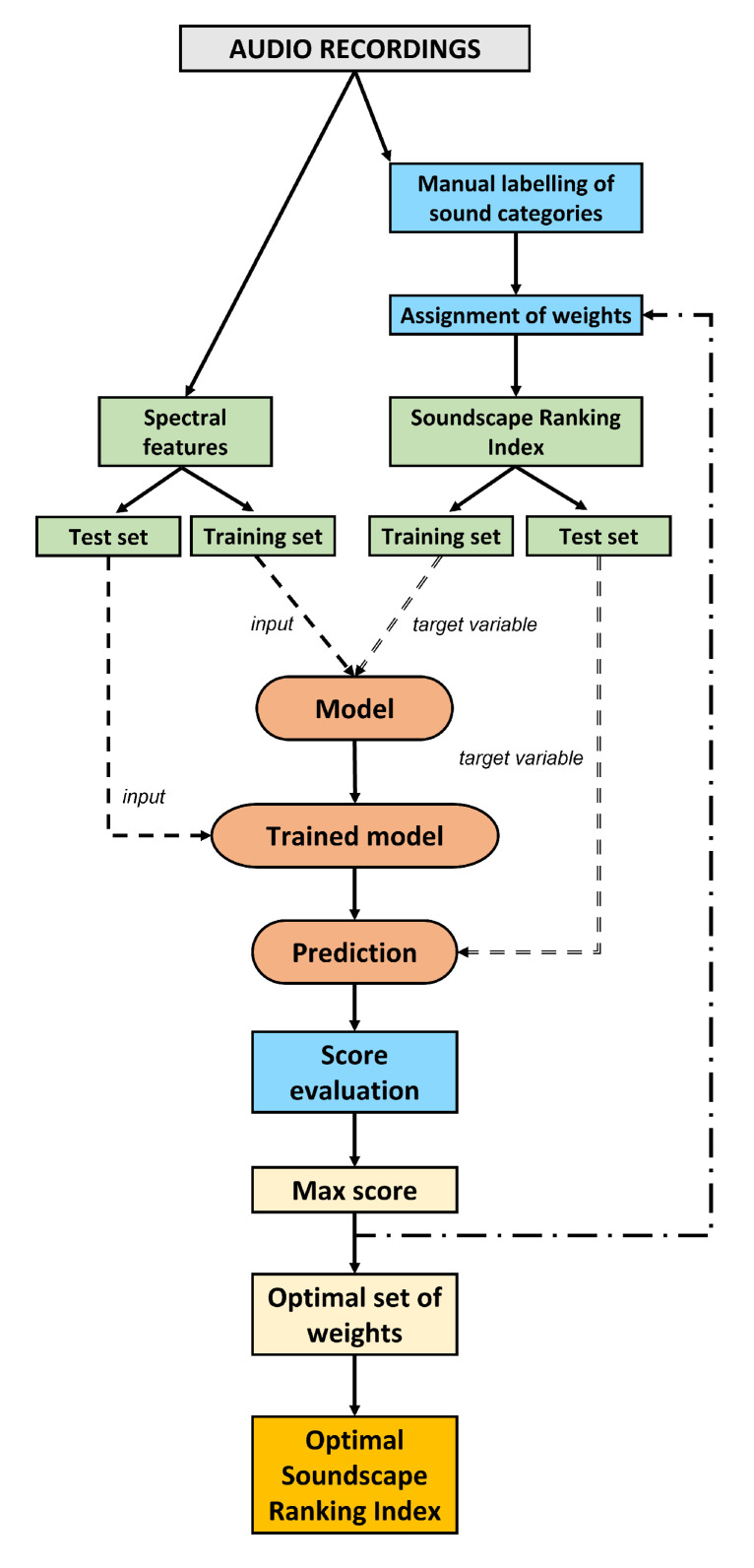
Scheme of the optimization procedure for the SRI according to the following steps: (1) assignment of weights to each sound category; (2) splitting of extracted spectral features and of the corresponding SRI into test and training sets; (3) running of classification models; (4) computation of classification score; (5) selection of optimal SRI according to the highest classification score.

Then, we calculated the spectral features of the sound recording and implemented the optimization scheme illustrated in Figure 4. In order to achieve this, we assumed that the coefficients $c_{N_{i}}$ can vary over the intervals reported in Table 1.

In order to proceed, both the spectral features and SRIs need to be split into “training” and “test” sets. The training set is used as the input for each classification model, whereas the performance of the test set is quantified according to the metrics described in Section 2.9. Here, we implicitly assumed that the optimal classification outcome produces the optimal separation/distance among the sites. This consideration comes from the analysis performed in a previous work where sites were clustered on the basis of distances (dissimilarities) calculated over the extracted spectral features of the audio files recorded in the area of study [51,52]. In the classification process, SRIs represent the target variable to be predicted by each model. This process is repeated for each combination of weights, $c_{N_{i}}$, where i>0, that is varied in the assigned interval. We considered a variation step Δc=0.1 for each of the four intervals for $c_{N_{i}}$, where the total number of choices is given by the product of the number of possible values for each coefficient $c_{N_{i}}$: 20 values for $c_{+}$ and $c_{-}$ each, and 30 for $c_{++}$ and $c_{--}$ each, yielding 20${}^{2}$ × 30${}^{2}$ = 360,000 combinations. The set of weights that define the optimal SRI was then obtained on the basis of a classification score as defined in Section 2.9.

### 2.7. Classification Models

In this section, we provide a brief description of the classification models used to predict the manual labeling sound categories, and thus the SRI index, from the ecoacoustic indices. In general, machine learning methods are better able to address multicollinearity issues and capture the potential non-linear relationships among variables. While binary classification is a more common application of them, they are also widely used for regressions when the target is continuous. In our case, we used classification algorithms for trinary (poor/medium/good) categories for determining the SRI.

The models taken into consideration in this study, which were implemented in Python programming language [53], are the following:

- Decision tree (DT);
- Random forest (RF);
- Support vector machine (SVM);
- Adaptive boosting (AdaBoost).

The supervised classification models implemented for the SRI optimization procedure were trained on the 80% of the data and tested on the remaining 20%. Data were split using a stratified procedure to keep the proportions between the classes of the corresponding target variable. Furthermore, class weights were used to take into account the class imbalance of the data set. In fact, training an algorithm with a skewed distribution of the classes can be achieved by giving different weights to both the majority and minority classes. The difference in weights will influence the classification of the classes during the training phase. The whole purpose is to penalize the misclassification made by the minority class by setting a higher class weight and, at the same time, reducing the weight for the majority class. The weight for the *j*th class of the target variable was chosen as follows:

(4) $$W_{j}=\frac{1}{\left|j\right|}\frac{T}{N},$$

where *T* is the total number of data items, *N* is the number of classes of the target variable, and |j| is the number of items in the jth class.

#### 2.7.1. Decision Trees

A decision tree (DT) is a non-parametric supervised learning method used to predict the value of a target variable by learning simple decision rules inferred from the data features [54,55]. DTs can be used to classify a set of data items using the inferred rules to recursively partition the feature space until each partition is pure or a stopping criterion is reached. Specifically, a DT learns a sequence of if-then statements, with each statement involving one feature and one split point. The topmost node in a DT is known as the root node and is constituted by the whole set of items. The root node is split starting from those variables that lead to the greatest degree of homogeneity.

Several measures were designed to evaluate the impurity of a partition, of which the Gini impurity (GI) is among the most popular ones [56]. Then, following the same criterion, each subsample, called a node, is split recursively into smaller nodes alongside single variables, and according to threshold values that identify two or more branches. Finally, when a node is no longer split into further nodes, either because a stopping criterion is reached or because it is pure, it becomes a leaf of the tree. An item is assigned to the class that has been associated with the leaf that it reaches.

#### 2.7.2. Random Forest

A random forest is a meta estimator that fits a number of decision tree classifiers on various sub-samples of the dataset, and uses averaging to improve the predictive accuracy and control overfitting. Random forests (RFs), or random decision forests, are an ensemble of learning methods used for classification, regression, and other tasks that operates by constructing a multitude of decision trees during the training procedure. For classification tasks, the output of the random forest is the class that is selected by most trees. In other words, it fits a number of decision tree classifiers on various sub-samples of the dataset and uses averaging to improve the predictive accuracy and control overfitting [57]. For this reason, RF generally outperforms decision tree models.

#### 2.7.3. Support Vector Machine

Support vector machines (SVMs) are supervised machine learning models that can be used for both classification and regression purposes [58,59]. They were initially devised as a binary classifier. SVMs map training data to points in space in order to maximize the width of the gap between the two categories. Thus, new data are mapped into that same space and predicted to belong to a category based on which side of the gap that they fall.

For multiclass classification, the same idea is employed by decomposing the multiclassification problem into multiple binary classification problems. This can be achieved by mapping data points to a high dimensional space to gain mutual linear separation between every two classes. This is called a *One-vs.-One* approach, which breaks down the multiclass problem into multiple binary classification problems using a binary classifier per each pair of classes. Another approach that can be used is the so-called *One-vs.-All* approach. In this case, a binary classifier per each class is used. The latter approach is used for the SRI optimization procedure.

In general, a data point is viewed as a *p*-dimensional vector (a list of *p* numbers) and we want to know whether we can separate such points with a (p−1)-dimensional hyperplane. This is called a linear classifier. There are many hyperplanes that might classify the data, but the goal of SVMs is to find the best hyperplane that represents the largest separation, or margin, between the classes. It is defined so that it is as far as possible from the closest data points from each of the classes. SVMs are effective in high-dimensional spaces even if the number of dimensions is greater than the number of samples. SVMs can efficiently perform a non-linear classification using what is called the kernel trick, implicitly mapping their inputs into high-dimensional feature spaces [60].

For the sake of clarity, let us consider a simple separable classification method in multidimensional space. Given two classes of examples clustered in feature space, any reasonable classifier hyperplane should pass between the means of the classes. One possible hyperplane is the decision surface that assigns a new point to the class whose mean is closer to it. This decision surface is geometrically equivalent to computing the class of a new point by checking the angle between two vectors: the vector connecting the two cluster means and the vector connecting the mid-point on that line with the new point. This angle can be formulated in terms of a dot product operation between vectors. The decision surface is implicitly defined in terms of the similarity between any new point and the cluster mean—a kernel function. This simple classifier is linear in the feature space whereas, in the input domain, it is represented by a kernel expansion in terms of the training examples.

Radial basis function (RBF) kernels are the most generalized form of kernelization and are one of the most widely used kernels due to its similarity to Gaussian distribution [61]. The RBF kernel function for two points *x* and *y* computes the similarity or how close they are to each other. This kernel can be mathematically represented as follows:

(5) $$K\left(x,y\right)=\exp\left(-\gamma \;||x-{y||}^{2}\right),$$

where γ is a hyperparameter that is inversely proportional to the standard deviation σ, and ||x−y|| is the Euclidean distance between two points *x* and *y*. The RBF kernel support vector machines are implemented using the scikit-learn library [62].

#### 2.7.4. AdaBoost

Adaptive boosting has been a very successful technique for solving two-class classification problems. It was first introduced in [63] with the AdaBoost algorithm. In going from two-class to multi-class classification, most boosting algorithms have been restricted to reducing the multi-class classification problem to multiple two-class problems, e.g., [63,64,65]. The natural multi-class extension of the two-class AdaBoost was obtained with the algorithm stagewise additive modeling using a multi-class exponential loss function (SAMME) [66].

The core principle of AdaBoost is to fit a sequence of weak learners (i.e., models that are only slightly better than random guessing, such as small decision trees) on repeatedly modified versions of the data. The predictions from all of them are then combined through a weighted majority vote (or sum) to produce the final prediction. The data modifications at each so-called boosting iteration consist of applying weights, ($w_{1},w_{2},\cdots ,w_{N}$), to each of the training samples. Initially, the weights are set to $w_{i}=1/N$ so that the first step simply trains a weak learner on the original data. For each successive iteration, the sample weights are individually modified and the learning algorithm is reapplied to the reweighted data. At a given step, those training examples that were incorrectly predicted by the boosted model induced at the previous step have their weights increased, whereas the weights are decreased for those that were predicted correctly. As iterations proceed, examples that are difficult to predict receive ever-increasing influence. Each subsequent weak learner is thereby forced to concentrate on the examples that are missed by the previous ones in the sequence [57].

### 2.8. Feature Extraction

In this section, we describe the features that were employed in the machine learning process. Feature extraction starts from the audio recordings and builds derived values (features) containing salient or summative information about the measured data. This process is intended to help the learning procedure by providing significant information about the content of the recordings. Here, we essentially used two types of features: those based on ecoacoustic indices and those based on mel-frequency cepstral coefficients (MFCCs) (see below).

#### 2.8.1. Ecoacoustic Indices

Ecoacoustic indices (ECOs) are generally used to quantify the soundscape in both marine and terrestrial habitats, and are grouped into categories aiming at quantifying the sound amplitude and its level of complexity and weighting the importance of geophonies, biophonies, and technophonies (soundscape). In this work, we focused on the following set of ecoacoustic indices:

- The acoustic entropy index (H) highlights the evenness of a signal’s amplitude over time and across the available range of frequencies [67].
- The acoustic complexity index (ACI) accounts for the modulation in intensity of a signal over changing frequencies [68].
- The normalized difference soundscape index (NDSI) accounts for the anthropogenic disturbance by computing the ratio between technophonies and biological acoustic signals [69].
- The bio-acoustic index (BI) is calculated as the area under the mean frequency spectrum above a threshold characteristic of the biophonic activity [15].
- The dynamic spectral centroid (DSC) indicates the center of mass of the spectrum [70].
- The acoustic diversity index (ADI) provides a measure of the local biodiversity at the community level without any species identification [70].
- The acoustic evenness index (AEI) provides reverse information of ADI with high values identifying recordings with the dominance of a narrow frequency band [70].

The ecoacoustic indices were calculated in the R environment (version 3.5.1 [36]). Specifically, the fast Fourier transform (FFT) was computed by the function *spectro* available in the R package “seewave” [71] in the frequency interval (0.1–12) kHz based on 1024 data points corresponding to a frequency resolution of FR = 46.875 Hz and, therefore, to a time resolution TR = 1/FR = 0.0213 s. The ecoacoustic indices were computed using the R package “soundecology” [72]. A dedicated script running in the “R” environment was written to calculate the DSC index. Two patterns of calculation were used:

- For each one-minute recording, we computed seven cumulative indices. Each recording is thus represented by seven features (seven indices).
- For each one-minute recording, we computed each index with a one-second time-step. Then, we calculated seven statistical descriptors (over 60 values): minimum, maximum, mean, median, skewness, kurtosis, and standard deviation. Each recording is thus represented by 49 features (seven indices times seven statistical descriptors).

Table 3 reports a summary of the extracted features employed in the classification process.

#### 2.8.2. Mel-Frequency Cepstral Coefficients (MFCCs)

The mel-frequency cepstrum (MFC) has become a convenient alternative for obtaining a reduced amount of data from each audio recording while keeping the core spectral information. The MFC is a representation of sound based on a linear discrete cosine transform (DCT) of a log-power spectrum on a non-linear MEL scale of frequency [73]. The latter is a perceptual scale of pitches judged by listeners to be equally spaced from one another (logarithmically distributed human auditory bands). Thus, after getting the spectrum onto the MEL scale, by applying filter banks and the logarithm of energies in each filter bank, the last step is to calculate the MFCCs [74]. This is carried out by fitting the cosines to the calculated log-energies using the DCT. MFCCs are the coefficients that collectively describe the MFC; that is, the amplitudes of the resulting spectrum. In most applications, the number of coefficients is twelve. This number represents a trade-off between an accurate description of the spectrum and dimensionality reduction of our feature space.

The calculation of the MFCCs was performed in the R environment using the default number of MEL filter banks; that is, 40 logarithmically distributed bands over the whole spectrum. Another important issue is the selection of the most convenient time window size for extracting the features of the data set. In this regard, we have to keep in mind that the dataset is obtained by computing a fixed number of features from an audio recording, usually referred to as a “window”.

A large time window size may capture relevant events but would result in a dataset with few instances. On the other hand, a small time window would result in a larger data set but may split the relevant events into several windows. For this reason, as we are trying to classify a summative description of the audio files (information described in Figure 3), we used a one-second time window as representative for distinguishing different sound characteristics. This number was selected to frame and window each audio file using a Hamming window with an overlap of 50%. In addition, in this case, we used two patterns of calculation (see Table 3):

- For each one-minute recording, we computed 12 MFCCs in a one-second time window. Then, we calculated seven statistical descriptors resulting from each audio recording: minimum, maximum, mean, median, skewness, kurtosis, and standard deviation. This corresponds to 84 features (12 MFCCs times 7 statistical descriptors).
- For each one-minute recording, we computed 12 MFCCs in a one-second time window. This corresponds to 1428 features (12 MFCCs times 119 time windows: 60 s window with 50% overlap).

### 2.9. Metrics

The performance of a model can be evaluated by the use of specific metrics that quantify the capability of the model to correctly predict one’s target. In our case, the performance of a model was evaluated based on a selection of the optimal set of weights, $c_{N_{i}}$, reported in Equation (2) and described in Table 2, which contributes to the definition of the SRI.

A confusion matrix is generally the starting point for calculating each metric. A confusion matrix is a table used to describe the performance of a classification model on a set of (training and test) data for which the true values are known. A strong discrepancy between the results obtained between training and test data may be indicative of an overfitting issue. It generally contains the following information: true positives, TPs, and true negatives, TN, which are the observations that are correctly predicted, and false positives, FP, and false negatives, FN, which occur when the actual class contradicts the predicted class. The derived metrics are the following [75]:

Precision: This represents the ratio of correctly predicted positive observations to the total predicted positive observations. A high precision is related to a low false positive rate:

(6) $$\mathrm{Precision}=\frac{\mathrm{TP}}{\mathrm{TP}+\mathrm{FP}}.$$

Recall (Sensitivity): This is the ratio of correctly predicted positive observations to all observations in the actual class. Thus, the recall tells us the proportion of correctly identified positives:

(7) $$\mathrm{Recall}=\frac{\mathrm{TP}}{\mathrm{TP}+\mathrm{FN}}.$$

F1-score: This is defined as the harmonic mean of precision and recall [75]. Therefore, the F1-score takes both FPs and FNs into account:

(8) $$F1\text{-}\mathrm{score}=\frac{2\cdot (\mathrm{Recall}\cdot \mathrm{Precision})}{\mathrm{Recall}+\mathrm{Precision}}.$$

This metric is useful in case both precision and recall are equally important. In our case, we decided to refer to the F1-score as the classification measure.

As a validation of the results, we used the k-fold cross-validation technique. It consists of an iterative procedure used to evaluate machine learning models. The procedure has a single parameter called k that refers to the number of folds that a given data sample has to be split into. This technique returns stratified folds; that is, folds obtained by preserving the percentage of samples for each class. At each kth iteration, the kth fold is used as the test set, whereas the other folds are used to train the model.

## 3. Results and Discussions

The results presented in this section refer to the audio files recorded on 25 May 2015, from 06:30 a.m. to 10:00 a.m. (CET). As described in Section 2.6, from the extracted features of all the audio recordings for which we had the corresponding labeling of sound categories, we ran four machine learning models to attempt a prediction of the soundscape ranking index calculated assigning a set of weights to each sound category. The range of variation in the above mentioned weights is reported in Table 1, and the best combination is calculated by the highest score provided by each classification model. The optimal set of weights are obtained by the highest classification measure, which, in our case, is the F1-score. Using the optimal set of weights, we calculated the SRI and derived a map of the environment sound quality of the area of study.

The machine learning algorithms selected usually work better on small data sets than deep neural networks. In fact, the latter require extremely large datasets to achieve high performances. Furthermore, a large dataset was not readily available and would be expensive and time-consuming to acquire. Another consideration when selecting classical machine learning algorithms concerns hyper-parameter tuning and the interpretability of these kinds of models. The underlying mechanisms of random forest, Adaboost, and SVMs are more straightforward than those of deep neural networks. Enhancing the interpretability also results in an easier tuning of hyper-parameters. However, for our preliminary study, we leaned on the default values as reported in [76], with the exception of the max depth parameter (used in DT and RF) used to control the size of the tree to prevent overfitting.

For each model, we split the entire dataset, consisting of 1220 audio files, into a training and test set with the following proportion: 80% of the dataset used for training and 20% of the dataset used for testing. As the reference measure to search for the optimal classification, we used the F1-score, which is more suited to our case, i.e., an uneven class distribution due to the limited sample size numerousness. Table 4 reports the results of the four models for each of the four extracted features.

In particular, the table contains the values of the weights and the corresponding classification measures. The weights can vary in an interval, meaning that the optimal classification measure (F1-score) can be obtained for a different combination of weights. The table also contains precision and recall as classification measures. All three measures are given in terms of the mean value ± standard deviation calculated over all classification classes defined by Equation (3).

In general, we can observe an increase in the classification performance as we provide more detailed information about the spectral content of each recording from 7 to 84 extracted features. For the 1428 features for each recording (1428 MFCCs), we observe a general drop (with the exception of the RF model) in the performance, more likely due to information redundancy contained in the time series. This redundancy is smoothed out by considering the statistical descriptors of the same time series (84 MFCCs).

A similar consideration can be carried out for the ecoacoustic indices. In this case, the 7 ECO features, derived by integrating the ecoacoustic indices over the whole length of the recording (1 minute), contain more condensed information; thus, it appears to not be enough to represent the complexity of the soundscape in a single summative index. On the other hand, the 49 ECO features provide a better representation of the spectral dynamics within each single audio recording. AdaBoost and RF models perform better. The AdaBoost model yields an F1-score of 0.70 (precision 0.65 and recall 0.78) with 84 MFCCs, and an F1-score of 0.69 (precision 0.68 and recall 0.70) with 49 ECO features. The RF model yields an F1-score of 0.71 (precision 0.71 and recall 0.78) with 84 MFCCs, and an F1-score of 0.70 (precision 0.68 and recall 0.75) with 1429 MFCCs. Hence, the RF model results in a slightly higher classification performance.

The highest metric ranking leads to the definition of nearly different sets of coefficients $c_{N_{i}}$ assigned to each sound category to be used in Equation (2). The DT model provides similar coefficients to the RF model. On the other hand, the SVM model gives the worst classification performance, and the resulting coefficients $c_{N_{i}}$ are completely discordant from the results of the other models.

### Discussions

The possibility of deriving an overall soundscape index summarizing the contribution of all the sound components and being able to rank them in terms of sound “quality” can be one of the empowering ecological tools used to help rapid on-site field and remote surveys. Here, we tested four extracted features (see Table 3) from recordings taken on 25 May 2015, from 06:30 a.m. to 10:00 a.m. (CET), referring to a measurement campaign over an area of approximately 22 hectares located in the Parco Nord of Milan.

**Figure 5:**
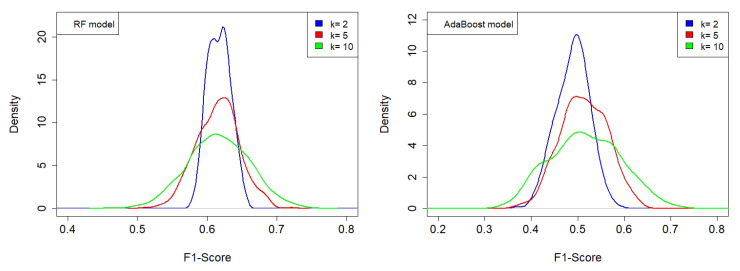
Kernel density distributions of F1-score for RF and AdaBoost, obtained using the k-fold splitting of the dataset for the following number of splits: k = (2, 5, 10), and repeating the operation 200, 100, and 100 times, respectively. (**Left panel**) The RF model with the following combination of weights: $c_{++}$ = 2.0, $c_{+}$ = 1.6, $c_{-}$ = −0.3, $c_{--}$ = −4.7. (**Right panel**) The AdaBoost model with the following combination of weights: $c_{++}$ = 2.9, $c_{+}$ = 2.0, $c_{-}$ = −1.7, $c_{--}$ = −2.5. RF model provides a more robust classification as its F1-score distribution presents maxima at higher values than for the AdaBoost model.

The idea of predicting an overall ranking index from a limited number of recordings, as initally defined in [51] and complemented in [52], was further developed in this paper, representing a first attempt to summarize the herd of ecoacoustic indices and spectral features for describing specific aspects of the audio-spectral content of a recording. This preliminary approach, based on ML techniques, revealed that two classification models, RF and AdaBoost, are able to provide rather good classification measures (F1-score = 0.70–0.71) using 84 extracted features from each recording. The two models are “evolutions” of DTs, of which AdaBoost required intensive time–machine calculation to complete the whole weight scan. In order to check for the possible overfitting of the models, we implemented a procedure called k-fold cross-validation, which refers to the number of groups, k, that a given data sample is to be split into. In this case, we tested the following values: k = (2, 5, 10), and repeated the operation 200, 100, and 100 times, respectively. Figure 5 illustrates the results in terms of the associated kernel density distributions.

We find that the RF model provides a more robust classification as its F1-score distribution presents maxima at higher values than for the AdaBoost model (see Table 5) for all the k-groups of split samples considered. As k increases, we also observe a spreading of the distribution due to a less numerous dataset.

**Figure 6:**
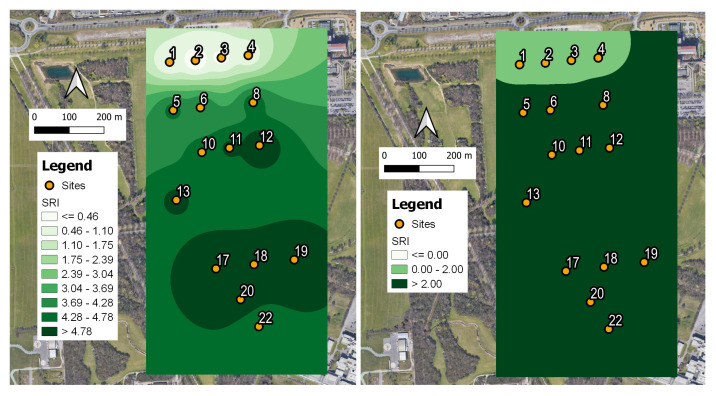
SRI map obtained using the following combination of weights as for the RF model: $c_{++}$ = 2.0, $c_{+}$ = 1.6, $c_{-}$ = −0.3, $c_{--}$ = −4.7. (**Left panel**) Range of continuous SRI variability: small differences in SRI at different sites are highlighted. (**Right panel**) Range of variability of SRI as defined by Equation (3): two main clusters are depicted, confirming previous analysis (see [50,52]). The legends indicate the ranges of variability of the SRI, and the sensor numbers correspond to the active ones as described in Figure 1.

In order to further validate the obtained results, we computed an SRI map over the study area based on the weights obtained for the RF model, which is reported in Table 4. For each of the 16 sites, we considered the median value of the SRI computed over all the measurements corresponding to the labeled recordings. The results are shown in Figure 6.

As expected, lower SRI values (poor/medium soundscape quality) are found close to the traffic noise sources (Sites 1–4), where the presence of higher traffic noise and less bird singing activity contribute significantly to this outcome. On the other hand, sites belonging to the park interior are less influenced by traffic noise and host higher biodiversity (many birds of different species singing). This result is reflected by the higher SRI values (good soundscape quality). Sites (5–8) are at intermediate positions and show a sort of transient behavior of SRI values (medium/good soundscape quality).

In Figure 6 (left panel), we show the actual continuously changing SRI values, whereas, in the right panel, they are selected as in Equation (3) to obtain a simplified picture. Both maps are fully compatible with the results obtained in [50], where the statistical analysis based on the computed ecoacoustic indices revealed the presence of a two-cluster separation, and also with a more recent estimation of the SRI based on a self-consistent statistical analysis [52]. The latter gives the optimized parameter values of $c_{++}$ = 2.29, $c_{+}$ = 0.766, $c_{-}$ = −1.528, and $c_{--}$ = −2.262, which are consistent with those reported in Figure 5 and Figure 6. Such a cluster separation is in agreement with the results of the aural survey (see Section 2.4) aimed at determining the sound components at the 16 sites (biophonies, technophonies, and geophonies).

## 4. Conclusions

The study of the soundscape within urban parks represents an increasingly important issue as they represent the link between natural habitats and highly populated urban areas. The evaluation of the soundscape is usually carried out through the help of ecoacoustics analysis and thus the use of well-known ecoacoustic indices. In this work, we gathered spectral information in the form of ecoacoustic indices and MFCCs to train four machine learning models to predict a single index (the soundscape ranking index, SRI) carrying information of different sound sources and, in addition, providing a soundscape ranking among different locations within the urban park.

The SRI has the advantage of yielding a quick overview of an environment given a set of extracted spectral features. We found that the seven statistical descriptors calculated for the 12 MFCCs (for a total of 84 features) are able to determine the optimal combination of weights that leads to a quite high classification score. Values for the F1-score of approximately 0.70 and 0.71 were obtained for AdaBoost and RF models, respectively. However, the RF model proved to be more robust when tested using the k-fold cross-validation procedure. Indeed, the information carried by the SRI represents a summative representation of the soundscape quality, which is essentially driven by the prevalence of the sound sources acting locally. As such, the SRI can be used to rapidly provide maps of environment sound quality on the basis of few audio recordings. The splitting of the SRI into three main intervals may somehow be adjusted by considering its quantization into smaller bins. This will allow us to obtain finer shades of the environment sound quality.

Mapping the SRI yielded similar results to those recently obtained in [50] through a simpler statistical approach and using a self-consistent SRI computation able to visualize the internal structure of the soundscape in the same habitat [52]. For these reasons, we may conclude that the SRI can become a useful tool for helping policy makers follow up the soundscape evolution in ‘‘natural” habitats within urban zones. More specifically, it can be employed, once fully developed, to evaluate the impact of noise-mitigating measures on ‘‘pocket” parks, urban parks, and residential redevelopment areas, thus allowing one to follow up the soundscape evolution in ‘‘natural” habitats within urban zones.

As already stated in the introduction, the availability of a small labeled dataset can undermine the performance of ML models, which represents a limitation of the present study. However, the obtained performance can be considered satisfactory and can represent a benchmark for future developments. As a future development of the present work, we envisage the use of larger labeled datasets. This can be achieved by using additional recordings with the corresponding aural survey, and/or via data augmentation using Monte Carlo techniques. We also envisage the application of NN models to develop more efficient classification schemes. In many situations, there is also the need to use different sound recorders to map extended areas simultaneously. Indeed, this procedure can introduce a bias in the analysis owing to different frequency responses of each sound recorder. This issue also needs to be addressed in our future works.

## Figures and Tables

**Table 1:** Range of variation in the coefficient $c_{N_{i}}$ assigned to each sound category, i=0,⋯,4, to be used in Equation (2). In this case, we aribitrarily chose $-5\leq c_{N_{i}}\leq 5$ for convenience.

$c_{N_{i}}$	Range	P(i) [52]
$c_{++}$	[2, 5]	P(1)
$c_{+}$	[0, 2]	P(2)
$c_{N_{0}}$	0	P(3)
$c_{-}$	[−2, 0]	P(4)
$c_{--}$	[−5, −2]	P(5)

**Table 2:** Coefficient $c_{N_{i}}$ assigned to each sound category to be used in Equation (2).

Category	Attribute	$c_{N_{i}}$
	no	$c_{N_{0}}$
Birds singing	few	$c_{+}$
	many	$c_{++}$
	no	$c_{N_{0}}$
Birds species	≲2	$c_{+}$
	>2	$c_{++}$
	0	0.00 × $c_{++}$
	(0, 25]	0.25 × $c_{++}$
Singing activity (%)	(25, 50]	0.50 × $c_{++}$
	(50, 75]	0.75 × $c_{++}$
	(75, 100]	1.00 × $c_{++}$
	no traffic	$c_{+}$
Traffic type	continuous	$c_{-}$
	intermittent	$c_{--}$
	zero	$c_{+}$
Traffic intensity	low	$c_{-}$
	high	$c_{--}$
Other sound sources	absent	$c_{N_{0}}$
	present	$c_{--}$

**Table 3:** Type of features extracted from the audio recording: characteristics and numerousness. Here, we use the abbreviations: ecoacoustic indices (ECOs), mel-frequency cepstral coefficients (MFCCs) (see also Section 2.8.2).

Type of Feature	Characteristics	Number of Features
Seven ecoacoustic indices (7 ECOs)	one-minute integration time	7
Seven ecoacoustic indices		
and	one-second integration time	49
seven statistical descriptors (49 ECOs)		
Twelve MFCCs		
and	one-second time window	84
seven statistical descriptors (84 MFCCs)		
Twelve MFCCs (1428 MFCCs)	one-second time window	1428

**Table 4:** Summary of results obtained for decision tree (DT), random forest (RF), support vector machine (SVM), adaptive boosting (AdaBoost) models, and four extracted features. Range of weights values and classification measures are reported. Precision, recall, and F1-score are provided with their standard deviations.

DT	$c_{++}$	$c_{+}$	$c_{-}$	$c_{--}$	Precision	Recall	F1-Score
(7 ECO)	[2.0, 2.1]	[1.9, 2.0]	[−1.2, −1.0]]	−3.6	0.64 ± 0.32	0.71 ± 0.10	0.62 ± 0.15
(49 ECO)	[2.3, 2.6]	[1.5, 1.7]	[−1.2, −1.0]	[−4.72, −4.0]	0.64 ± 0.26	0.67 ± 0.03	0.63 ± 0.13
(84 MFCC)	[2.0, 2.3]	[1.8, 2.0]	[−1.2, −1.0]	[−4.4, −3.9]	0.68 ± 0.24	0.73 ± 0.09	0.68 ± 0.10
(1428 MFCC)	[2.5, 2.6]	[1.4, 1.5]	−1.0	[−5.0, −4.8]	0.63 ± 0.22	0.64 ± 0.12	0.62 ± 0.12
**RF**							
(7 ECO)	2.0	2.0	[−1.5, −1.4]	[−2.6, −2.7]	0.63 ± 0.28	0.75 ± 0.16	0.63 ± 0.12
(49 ECO)	2.0	1.6	−0.3	−4.7	0.70 ± 0.28	0.78 ± 0.14	0.69 ± 0.12
(84 MFCC)	[2.0, 2.1]	[0.7, 0.8]	−0.1	[−4.3, −4.1]	0.71 ± 0.22	0.78 ± 0.15	0.71 ± 0.15
(1428 MFCC)	2.0	2.0	−1.7	−2.8	0.68 ± 0.15	0.75 ± 0.16	0.70 ± 0.03
**SVM**							
(7 ECO)	2.5	1.6	−1.0	−4.7	0.60 ± 0.18	0.60 ± 0.06	0.59 ± 0.10
(49 ECO)	[4.3, 5.0]	[1.9, 2.0]	[−1.2, −1.0]	[−2.3, −2.0]	0.33 ± 0.58	0.33 ± 0.56	0.33 ± 0.47
(84 MFCC)	[4.3, 5.0]	[1.9, 2.0]	[−1.2, −1.0]	[−2.3, −2.0]	0.33 ± 0.58	0.33 ± 0.56	0.33 ± 0.47
(1428 MFCC)	[4.3, 5.0]	[1.8, 2.0]	[−1.2, −1.0]	[−2.3, −2.0]	0.33 ± 0.58	0.33 ± 0.56	0.33 ± 0.47
**AdaBoost**							
(7 ECO)	[2.4, 2.6]	[1.3, 1.7]	[−1.8, −1.5]	[−2.2, −2.0]	0.60 ± 0.19	0.64 ± 0.18	0.62 ± 0.15
(49 ECO)	[2.3, 2.4]	2.0	[−1.8, −1.7]	[−3.4, −3.2]	0.68 ± 0.05	0.70 ± 0.05	0.69 ± 0.02
(84 MFCC)	2.9	2.0	−1.7	−2.5	0.65 ± 0.24	0.78 ± 0.15	0.70 ± 0.14
(1428 MFCC)	[2.0, 2.4]	[1.5, 1.8]	[−1.2, −1.0]	[−4.1, −3.3]	0.66 ± 0.09	0.68 ± 0.04	0.67 ± 0.04

**Table 5:** Mean F1-score ± standard deviation calculated for the distributions illustrated in Figure 5: random forest (RF) and adaptive boosting (AdaBoost) models, where k = (2, 5, 10).

k	RF	AdaBoost
2	0.62 ± 0.02	0.49 ± 0.04
5	0.62 ± 0.03	0.51 ± 0.05
10	0.62 ± 0.04	0.52 ± 0.08

## Data Availability

Data are available upon request.

## References

[1] Dumyahn S.L., Pijanowski B.C. (2011). Soundscape conservation. Landsc. Ecol..

[2] Schafer R.M. (1993). The Soundscape: Our Sonic Environment and the Tuning of the World.

[3] Barber J.R., Crooks K.R., Fristrup K.M. (2010). The costs of chronic noise exposure for terrestrial organisms. Trends Ecol. Evol..

[4] Doser J.W., Hannam K.M., Finley A.O. (2020). Characterizing functional relationships between technophony and biophony: A western New York soundscape case study. Landsc. Ecol..

[5] Francis C.D., Newman P., Taff B.D., White C., Monz C.A., Levenhagen M., Petrelli A.R., Abbott L.C., Newton J., Burson S. (2017). Acoustic environments matter: Synergistic benefits to humans and ecological communities. J. Environ. Manag..

[6] Lawson G.M., Qun F., Brearley J. (2011). Networks cities and ecological habitats. Networks Cities.

[7] Sueur J., Farina A., Gasc A., Pieretti N., Pavoine S. (2014). Acoustic indices for biodiversity assessment and landscape investigation. Acta Acust. United Acust..

[8] Krause B. (2002). The Loss of Natural Soundscapes. Earth Isl. J..

[9] Pijanowski B.C., Farina A., Gage S.H., Dumyahn S.L., Krause B.L. (2011). What is soundscape ecology? An introduction and overview of an emerging new science. Landsc. Ecol..

[10] Pavan G., Farina A., Gage S.H. (2017). Fundamentals of Soundscape Conservation. Ecoacoustics: The Ecological Role of Sounds.

[11] Sethi S.S., Jones N.S., Fulcher B.D., Picinali L., Clink D.J., Klinck H., Orme C.D.L., Wrege P.H., Ewers R.M. (2020). Characterizing soundscapes across diverse ecosystems using a universal acoustic feature set. Proc. Natl. Acad. Sci. USA.

[12] Lellouch L., Pavoine S., Jiguet F., Glotin H., Sueur J. (2014). Monitoring temporal change of bird communities with dissimilarity acoustic indices. Methods Ecol. Evol..

[13] Kasten E.P., Gage S.H., Fox J., Joo W. (2012). The remote environmental assessment laboratory’s acoustic library: An archive for studying soundscape ecology. Ecol. Inform..

[14] Eldridge A., Guyot P., Moscoso P., Johnston A., Eyre-Walker Y., Peck M. (2018). Sounding out ecoacoustic metrics: Avian species richness is predicted by acoustic indices in temperate but not tropical habitats. Ecol. Indic..

[15] Boelman N.T., Asner G.P., Hart P.J., Martin R.E. (2007). Multitrophic invasion resistance in hawaii: Bioacoustics, field surveys, and airborne remote sensing. Ecol. Appl..

[16] Benocci R., Brambilla G., Bisceglie A., Zambon G. (2020). Eco-Acoustic Indices to Evaluate Soundscape Degradation Due to Human Intrusion. Sustainability.

[17] Bertucci F., Parmentier E., Berten L., Brooker R.M., Lecchini D. (2015). Temporal and spatial comparisons of underwater sound signatures of different reef habitats in Moorea Island, French Polynesia. PLoS ONE.

[18] Harris S.A., Shears N.T., Radford C.A. (2016). Ecoacoustic indices as proxies for biodiversity on temperate reefs. Methods Ecol. Evol..

[19] Pérez-Granados C., Traba J. (2021). Estimating bird density using passive acoustic monitoring: A review of methods and suggestions for further research. Ibis.

[20] Shonfield J., Bayne E.M. (2017). Autonomous recording units in avian ecological research: Current use and future applications. Avian Conserv. Ecol..

[21] Benocci R., Roman H.E., Bisceglie A., Angelini F., Brambilla G., Zambon G. (2021). Eco-acoustic assessment of an urban park by statistical analysis. Sustainability.

[22] LeCun Y., Boser B., Denker J.S., Henderson D., Howard R.E., Hubbard W., Jackel L.D. (1989). Backpropagation applied to handwritten zip code recognition. Neural Comput..

[23] Lewis J.P. Creation by refinement: A creativity paradigm for gradient descent learning networks. Proceedings of the IEEE 1988 International Conference on Neural Networks.

[24] Todd P.M., Touretzky D., Hinton G., Sejnowski T. (1988). A sequential neural network design for musical applications. 1988 Connectionist Models Summer School.

[25] Cavallari G.B., Ribeiro L.S., Ponti M.A. (2018). Unsupervised representation learning using convolutional and stacked auto-encoders: A domain and cross-domain feature space analysis. Proceedings of the 31st SIBGRAPI Conference on Graphics, Patterns and Images (SIBGRAPI).

[26] Ponti M.A., Ribeiro L.S.F., Nazare T.S., Bui T., Collomosse J. (2017). Everything you wanted to know about deep learning for computer vision but were afraid to ask. Proceedings of the 30th SIBGRAPI Conference on Graphics, Patterns and Images Tutorials (SIBGRAPI-T).

[27] Christin S., Hervet É., Lecomte N. (2019). Applications for deep learning in ecology. Methods Ecol. Evol..

[28] Fairbrass A.J., Firman M., Williams C., Brostow G.J., Titheridge H., Jones K.E. (2019). CityNet–Deep learning tools for urban ecoacoustic assessment. Methods Ecol. Evol..

[29] Lin T.H., Tsao Y. (2020). Source separation in ecoacoustics: A roadmap towards versatile soundscape information retrieval. Remote Sens. Ecol. Conserv..

[30] Navarro J.M., Pita A. (2023). Machine Learning Prediction of the Long-Term Environmental Acoustic Pattern of a City Location Using Short-Term Sound Pressure Level Measurements. Applied Sciences.

[31] Orga F., Socoró J.C., Alías F., Alsina-Pagès R.M., Zambon G., Benocci R., Bisceglie A. Anomalous Noise Events Considerations for the Computation of Road Traffic Noise Levels: The DYNAMAP’s Milan Case Study. Proceedings of the 24th International Congress on Sound and Vibration, ICSV 2017.

[32] Piczak K.J. (2015). Environmental sound classification with convolutional neural networks. Proceedings of the IEEE 25th International Workshop on Machine Learning for Signal Processing (MLSP).

[33] Salamon J., Bello J.P. (2017). Deep convolutional neural networks and data augmentation for environmental sound classification. IEEE Signal Process. Lett..

[34] Ward J.H. (1963). Hierarchical grouping to optimize an objective function. J. Am. Stat. Assoc..

[35] Ruff Z.J., Lesmeister D.B., Appel C.L., Sullivan C.M. (2021). Workflow and convolutional neural network for automated identification of animal sounds. Ecol. Indic..

[36] R Core Team (2018). R: A Language and Environment for Statistical Computing.

[37] Vidaña-Vila E., Navarro J., Stowell D., Alsina-Pagès R.M. (2021). Multilabel Acoustic Event Classification Using Real-World Urban Data and Physical Redundancy of Sensors. Sensors.

[38] Mullet T.C., Gage S.H., Morton J.M., Huettmann F. (2016). Temporal and spatial variation of a winter soundscape in south-central Alaska. Landsc. Ecol..

[39] Quinn C.A., Burns P., Gill G., Baligar S., Snyder R.L., Salas L., Goetz S.J., Clark M.L. (2022). Soundscape classification with convolutional neural networks reveals temporal and geographic patterns in ecoacoustic data. Ecol. Indic..

[40] Giannakopoulos T., Siantikos G., Perantonis S., Votsi N.E., Pantis J. Automatic soundscape quality estimation using audio analysis. Proceedings of the 8th ACM International Conference on Pervasive Technologies Related to Assistive Environments.

[41] Tsalera E., Papadakis A., Samarakou M. (2020). Monitoring, profiling and classification of urban environmental noise using sound characteristics and the KNN algorithm. Energy Rep..

[42] Lojka M., Pleva M., Kiktová E., Juhár J., Čižmár A. (2014). Ear-tuke: The acoustic event detection system. Proceedings of the Multimedia Communications, Services and Security: 7th International Conference, MCSS 2014.

[43] Pita A., Rodriguez F.J., Navarro J.M. (2021). Cluster analysis of urban acoustic environments on Barcelona sensor network data. Int. J. Environ. Res. Public Health.

[44] Pita A., Rodriguez F.J., Navarro J.M. (2022). Analysis and Evaluation of Clustering Techniques Applied to Wireless Acoustics Sensor Network Data. Appl. Sci..

[45] Luitel B., Murthy Y.S., Koolagudi S.G. Sound event detection in urban soundscape using two-level classification. Proceedings of the 2016 IEEE Distributed Computing, VLSI, Electrical Circuits and Robotics (DISCOVER).

[46] Maijala P., Shuyang Z., Heittola T., Virtanen T. (2018). Environmental noise monitoring using source classification in sensors. Appl. Acoust..

[47] Ye J., Kobayashi T., Murakawa M. (2017). Urban sound event classification based on local and global features aggregation. Appl. Acoust..

[48] Gómez-Gómez J., Vidaña-Vila E., Sevillano X. (2023). Western editerranean wetland birds dataset: A new annotated dataset for acoustic bird species classification. Ecol. Inform..

[49] Brambilla G., Confalonieri C., Benocci R. (2019). Application of the intermittency ratio metric for the classification of urban sites based on road traffic noise events. Sensors.

[50] Benocci R., Potenza A., Bisceglie A., Roman H.E., Zambon G. (2022). Mapping of the Acoustic Environment at an Urban Park in the City Area of Milan, Italy, Using Very Low-Cost Sensors. Sensors.

[51] Benocci R., Roman H.E., Bisceglie A., Angelini F., Brambilla G., Zambon G. (2022). Auto-correlations and long time memory of environment sound: The case of an Urban Park in the city of Milan (Italy). Ecol. Indic..

[52] Benocci R., Afify A., Potenza A., Roman H.E., Zambon G. (2023). Self-consistent Soundscape Ranking Index: The Case of an Urban Park. Sensors.

[53] Python. https://www.python.org/.

[54] Kamiński B., Jakubczyk M., Szufel P. (2018). A framework for sensitivity analysis of decision trees. Cent. Eur. J. Oper. Res..

[55] Quinlan J.R. (1987). Simplifying decision trees. Int. J. Man-Mach. Stud..

[56] Jost L. (2006). Entropy and diversity. Oikos.

[57] Hastie T., Friedman J.H., Tibshirani R. (2009). The Elements of Statistical Learning: Data Mining, Inference, and Prediction.

[58] Cortes C., Vapnik V. (1995). Support-vector networks. Mach. Learn..

[59] Support Vector Machines. Https://scikit-learn.org/stable/modules/svm.html.

[60] Aizerman M.A., Braverman E.M., Rozonoer L.I. (1964). Theoretical foundations of the potential function method in pattern recognition learning. Autom. Remote Control.

[61] Radial Basis Function Kernel. Https://en.wikipedia.org/wiki/Radial_basis_function_kernel.

[62] Scikit-Learn Implementation of SVM. https://scikit-learn.org/stable/auto_examples/svm/plot_rbf_parameters.html.

[63] Freund Y., Schapire R.E. (1997). A decision-theoretic generalization of on-line learning and an application to boosting. J. Comput. Syst. Sci..

[64] Schapire R. (1997). Using output codes to boost multiclass learning problems. Proceedings of the Fourteenth International Conference on Machine Learning.

[65] Schapire R.E., Singer Y. Improved boosting algorithms using confidence-rated predictions. Proceedings of the Eleventh Annual Conference on Computational Learning Theory.

[66] Hastie T., Rosset S., Zhu J., Zou H. (2009). Multi-class adaboost. Stat. Its Interface.

[67] Sueur J., Pavoine S., Hamerlynck O., Duvail S. (2008). Rapid acoustic survey for biodiversity appraisal. PLoS ONE.

[68] Pieretti N., Farina A., Morri D. (2011). A new methodology to infer the singing activity of an avian community: The Acoustic Complexity Index (ACI). Ecol. Indic..

[69] Grey J.M., Gordon J.W. (1978). Perceptual effects of spectral modifications on musical timbres. J. Acoust. Soc. Am..

[70] Yang W., Kang J. (2005). Soundscape and sound preferences in urban squares: A case study in Sheffield. J. Urban Des..

[71] Seewave: Sound Analysis and Synthesis. https://cran.r-project.org/web/packages/seewave/index.html.

[72] Soundecology: Soundscape Ecology. https://cran.r-project.org/web/packages/soundecology/index.html.

[73] Davis S., Mermelstein P. (1980). Comparison of parametric representations for monosyllabic word recognition in continuously spoken sentences. IEEE Trans. Acoust. Speech Signal Process..

[74] Sahidullah M., Saha G. (2012). Design, analysis and experimental evaluation of block based transformation in MFCC computation for speaker recognition. Speech Commun..

[75] Precision-Recall. https://scikit-learn.org/stable/auto_examples/model_selection/plot_precision_recall.html.

[76] Supervised Learning. https://scikit-learn.org/stable/supervised_learning.html#supervised-learning.

